# The influence of digital platforms on gig workers: A systematic literature review

**DOI:** 10.1016/j.heliyon.2024.e41491

**Published:** 2024-12-26

**Authors:** Farah Diba Almayanda Alauddin, Aini Aman, Mohd Fahmi Ghazali, Sity Daud

**Affiliations:** a Faculty of Economics and Management, Universiti Kebangsaan Malaysia, Malaysia; b School of International Studies, Universiti Utara Malaysia, Malaysia; c Center for Global Business & Digital Economy Studies, Faculty of Economics and Management, Universiti Kebangsaan Malaysia, Malaysia; d Research Center for History, Politics, and International Affairs, Faculty of Social Sciences and Humanity, Universiti Kebangsaan Malaysia, Malaysia

**Keywords:** Gig economy, Digital platforms, Gig workers, Systematic review, PRISMA, Sustainable economic development

## Abstract

Digital platforms have significantly transformed the labor market, particularly in the gig economy. Despite this issue's growing importance, no systematic literature review has explicitly examined the influence of digital platforms on gig workers. This study fills this gap by conducting a comprehensive review of 18 articles out of 81 published between 2019 and 2023, retrieved from the Scopus and Web of Science databases, using the PRISMA framework. Thematic analysis revealed 12 key themes that capture the complex influence of digital platforms on gig workers, suggesting that platforms offer both opportunities and challenges. Digital platforms provide low barriers to entry, facilitate task allocation, and offer flexible work arrangements. However, gig workers face significant challenges, such as a lack of social protections, algorithmic control, intense competition, and downward pressure on wages. The implications are significant for sustainable economic development in the platform economy, underscoring the importance of collective organizing, re-evaluating platform practices, and strengthening labor regulations. As the gig economy expands globally, researchers, platforms, and policymakers must work together to ensure that the benefits of digital platforms are shared more equitably and that gig workers can thrive in this new world of work. Given the scarcity of papers published about this topic, the study has no specific focus on region and industry. In addition, this study only extracts papers that are published in English.

## 1 Introduction

**Fig. 1:**
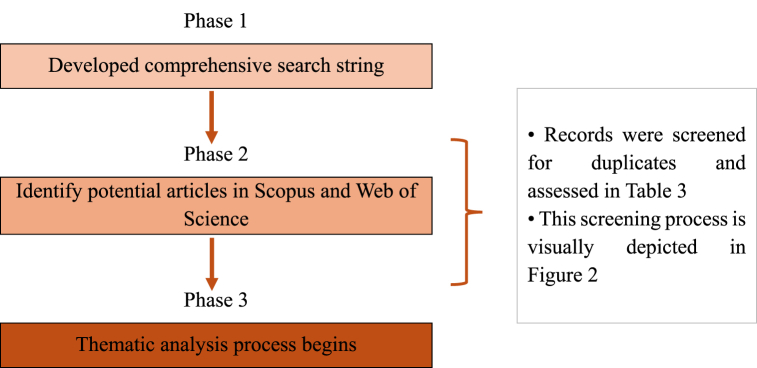
Flow diagram (adapted from [19]).

**Fig. 2:**
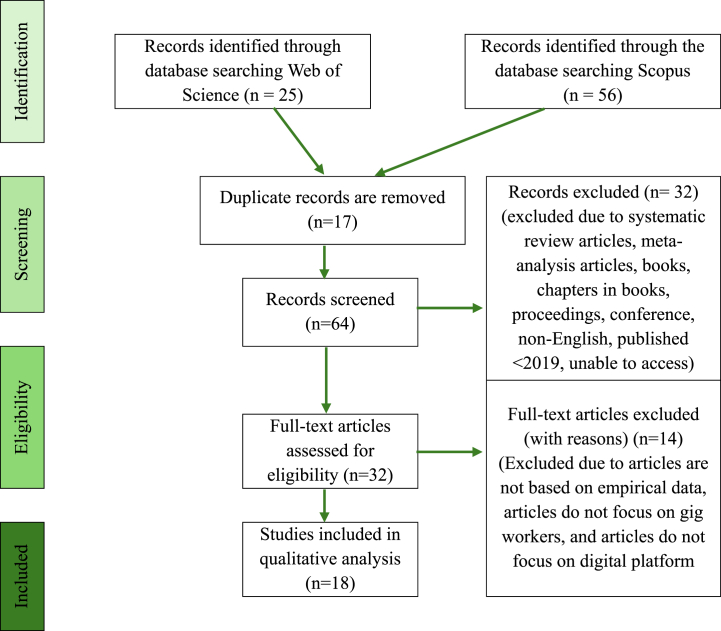
Flow diagram of the study (adapted from [[19], [24]]).

**Fig. 3:**
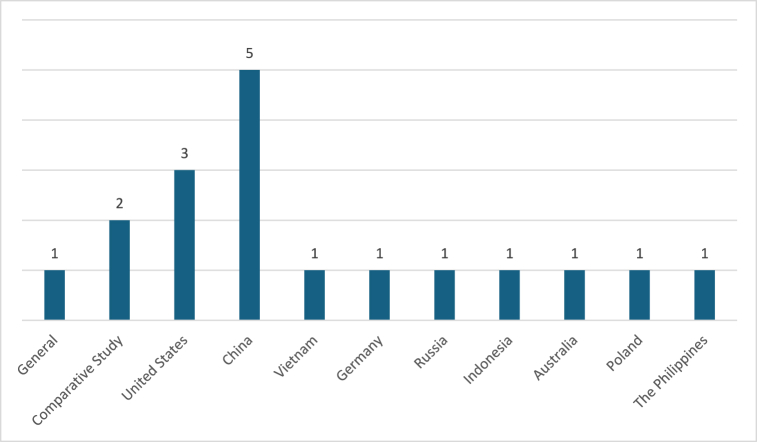
Countries where the studies were conducted.

**Fig. 4:**
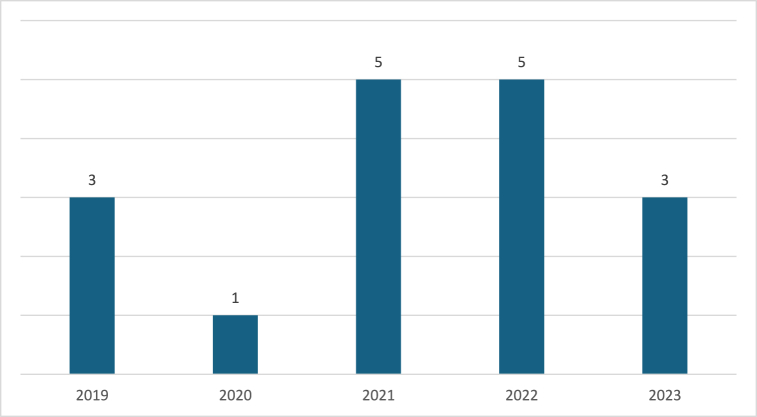
Year of publication.

**Fig. 5:**
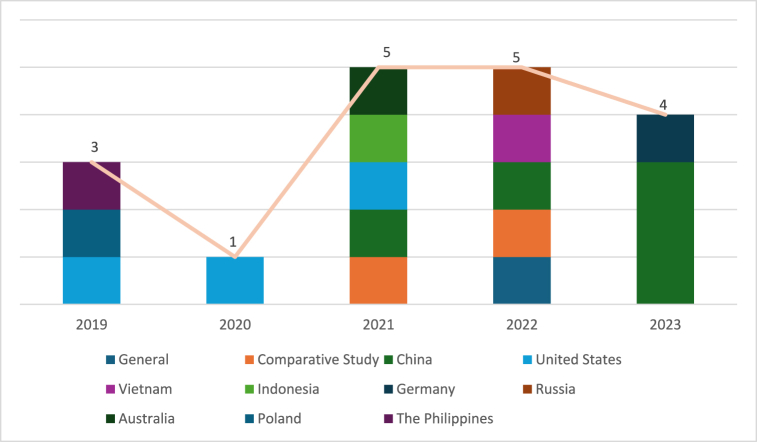
Countries where the studies were conducted and year of publication.

**Fig. 6:**
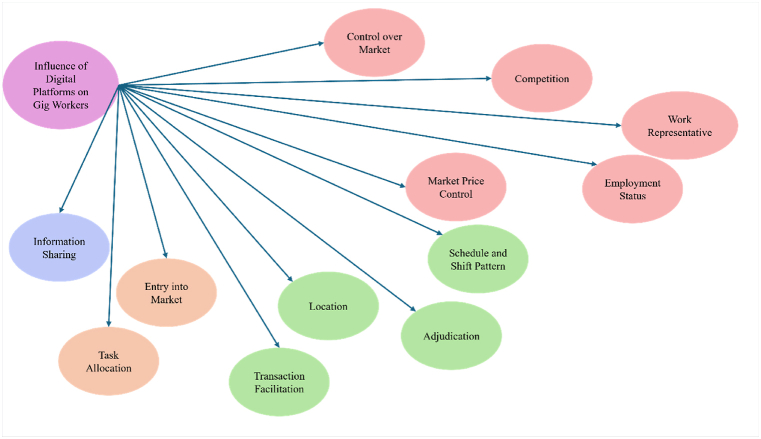
Cluster chart for major theme.

The rise of the gig economy has profoundly reshaped traditional labor practices, with an increasing prevalence of temporary, flexible, and on-demand work arrangements [1]. This shift has created a new class of gig workers who typically engage in short-term tasks or projects [2]. While the concept of gig work itself is not entirely novel, digital platforms have played a pivotal role in the rapid global expansion of the gig workforce [3]. As the gig economy grows, digital platforms are becoming increasingly influential in connecting gig workers with opportunities and clients, significantly impacting their working conditions, earning potential, and overall livelihoods (see Fig. 1, Fig. 2, Fig. 3, Fig. 4, Fig. 5, Fig. 6 [[19], [24]]).

Historically, gig work referred to short-term contracts and freelance work distinct from permanent employment [3]. However, the definition has evolved with the introduction of advanced technologies such as blockchain, smart contracts, and AI [4]. Digital platforms have transformed the nature of gig work, offering gig workers greater autonomy and flexibility in task selection [[5], [6], [7]], shorter recruitment periods [8], and lower barriers to entry, enabling participation from diverse backgrounds [9]. However, gig workers also face unique challenges, such as job insecurity due to uncertainty in task allocation and demand [10], and for remote gig workers, social isolation, intense working schedules, and prolonged periods of social alienation [4].

Gig workers are often classified as contractors or self-employed individuals; they are frequently excluded from the incentives and benefits traditionally provided to conventional workers and may experience income insecurity, deteriorating working conditions, and uncertain social protections [11]. Digital platforms enable gig workers to find suitable employment opportunities while engaged in other forms of work, avoid traditional recruitment agencies that charge fees, and access work as migrant workers [12]. However, it is essential to note that not all gig work is connected to the platform company. In some cases, the digital platform serves only as a mechanism for platform companies and gig workers to connect [13].

The gig economy has experienced substantial growth on a global scale in recent years. Wood et al. [14] reported that nearly 70 million workers registered on platform apps in 2019, with the number of users on online platforms increasing at an annual rate of 26 %. By 2021, there were approximately 163 million profiles of registered workers on digital platforms globally [15]. The World Bank [16] estimates that the global online gig workforce ranges between 154 million and 435 million individuals, constituting 4.4 %–12.5 % of the global labor force. This surge underscores the growing demand for flexible, digitally connected work arrangements, as evidenced by 545 online gig platforms worldwide. In Malaysia, the gig economy has similarly expanded. According to the Malaysia Digital Economy Corporation (MDEC) in its 2024 Budget Digital Economy Snapshot, the local gig economy is valued at approximately RM 1.61 billion. Local platforms are expected to grow from RM 371.4 million in 2021 to RM 650 million by 2025 [17]. About 1.12 million individuals are engaged in the gig industry in Malaysia, which is experiencing an annual growth rate of 23 % [17].

This systematic review aims to synthesize evidence on the key ways digital platforms influence gig workers, guided by the overarching research question: What are the main influences of digital platforms on gig workers? The review's findings have important implications for sustainable and inclusive economic development in the digital age, as policymakers, platforms, and researchers grapple with how to ensure the benefits of technological innovation in the platform economy are shared more equitably.

### 1.1 The need for systematic review

A systematic review is a rigorous quantitative and qualitative process of identifying, combining, and evaluating all available data to produce an empirically determined response to a research question [18,19]. Systematic reviews offer several advantages over conventional literature reviews, including a more thorough article retrieval process, a broader research scope, and more significant objectives that can help control research bias [19].

Given the relatively new nature of research on digital platforms and gig workers, there have been limited systematic literature review articles on this topic, where recent systematic reviews have only explored related issues such as the challenges facing HR in the platform economy [20], factors shaping online labor markets [21], and the physical and psychological experience of gig work [22], to the best of the researcher's knowledge, there has not yet been a systematic review explicitly examining the influence of digital platforms on gig workers. This review addresses that gap by focusing on the most recent and relevant research published between 2019 and 2023, drawing on the comprehensive Scopus and Web of Science databases.

This paper seeks to fill the gap by systematically reviewing the relevant literature to examine the evidence on the influence of digital platforms on gig workers. The study is important due to the lack of existing research that scrutinizes the impact of digital platforms on gig workers, especially considering that gig workers existed before the advent of digital platforms. The study is also significant because it provides in-depth information on the review procedures adopted, including keyword identification, article screening, article eligibility, and databases used, thereby avoiding duplication of research or analysis by future researchers.

The main research question guiding this systematic literature review is: What are the influences of digital platforms on gig workers? The focus is on the impact experienced by gig workers as they engage with digital platforms. The paper has no specific geographical or industry focus, as limited research has been conducted on this topic to the researcher's knowledge. The following section discusses the approach utilized to answer the research question, while the third section conducts a systematic review and synthesizes the scientific literature to identify, select, and evaluate significant research on the influences of digital platforms on gig workers. The final section discusses measures that future scholars should consider when conducting studies in this area.

## 2 Materials and methods

This section presents the five main sub-sections: PRISMA, resources, inclusion and exclusion criteria, systematic review process, and data abstraction and analysis.

### 2.1 PRISMA

Preferred Reporting Items for Systematic Reviews and Meta-Analysis (PRISMA) is a published standard for systematic literature reviews. The publication standards guide researchers with the necessary information to evaluate and examine the quality of a review [19]. PRISMA is particularly suitable for management because it clearly defines the research question and identifies a particular research's inclusion and exclusion criteria [23]. Additionally, PRISMA facilitates the examination of an extensive scientific literature database at a defined time, allowing for an accurate search of terms related to the influence of digital platforms on gig workers.

### 2.2 Resources

This study's review methods used two central databases: Web of Science (Social Science Citation Indexed) and Scopus. These databases are considered robust and cover more than 256 fields of study, including research on gig workers and digital platforms. The keyword "digital platforms" alone resulted in 28,944 documents in Scopus and 2367 in Web of Science. In comparison, the term "gig workers" accounted for 1516 documents in Scopus and 203 documents in Web of Science. Digital platforms and gig workers are rigorously discussed from numerous perspectives, making these databases the primary resources for articles to be reviewed in this systematic literature review.

### 2.3 The systematic review process for selecting the articles

The systematic review process for selecting relevant articles for this study consisted of three main stages: identification, screening, and eligibility (see Table 1 (adapted from [19])).

#### 2.3.1 Identification

The first stage involved identifying keywords and searching for related or similar terms based on thesauruses, dictionaries, encyclopedias, and past research. The Scopus and Web of Science search string in Table 2 was developed in July 2023 after all relevant keywords were determined and finalized. In total, this study successfully retrieved a total of 81 articles from both databases.

**Table 1:** Development of keywords for search strings.

Keywords	Synonyms	Related Terms	Variations
Gig workers	On-demand workers, freelancers, gig labor	Online labor, precarious worker, temporary worker	Gig worker, online labor, gig labor
Digital Platforms	On-demand platforms	Crowdsourcing platform, Online platform, Platform companies	Digital platform
Influence	Effect, impact		Influencing, influent

**Table 2:** The search strings.

Databases	Keywords used
Web of Science	((“gig work∗” OR “on-demand work∗” OR “freelance∗” OR “gig labo∗” OR “online labo∗” OR “precarious work∗” OR “temporary work∗”) AND (“on-demand platform∗” OR “crowdsourcing platform∗” OR “online platform∗” OR “platform compan∗”) AND (“influence∗” OR “influent” OR “effect∗” OR “impact∗”))
Scopus	TITLE-ABS-KEY((“gig work∗” OR “on-demand work∗” OR “freelance∗” OR “gig labo∗” OR “online labo∗” OR “precarious work∗” OR “temporary work∗”) AND (“on-demand platform∗” OR “crowdsourcing platform∗” OR “online platform∗” OR “platform compan∗”) AND (“influence∗” OR “influent” OR “effect∗” OR “impact∗”))

#### 2.3.2 Screening

The screening stage was conducted to remove duplicate articles and assess the remaining articles against pre-defined exclusion and inclusion criteria [19]. The results of WOS and Scopus were merged manually when the results were listed in Microsoft Excel, and overlapping results with similar authors’ names, titles of the articles, abstracts, and publication platforms were excluded. The authors used the manual matching and deduplication method. The advantages of this method are that the results are more accurate due to human judgment, better context understanding, and flexible decision-making. In the first stage of screening, a total of 17 duplicate articles were excluded. In the second stage, 32 articles were screened based on several criteria determined by the researcher (Table 3). The first criterion was the type of literature, focusing only on journals and research articles as the primary source of empirical data. Systematic reviews, books, chapters, and conference papers were excluded because these publications act as secondary sources. Seven papers were excluded because they could not be accessed because of restriction and permission since the university did not subscribe to these papers; hence researcher could not access the paper even after requesting from the authors. Additionally, the review only focused on articles published in English and within the timeline range of 2019-2023. Overall, 32 papers were excluded in this stage.

**Table 3:** Criteria, inclusion, and exclusion of articles.

Criterion	Eligibility	Exclusion
Literature Type	Journal/Articles	Journals (systematic review), book, chapter in book, conference proceedings, unable to access
Accessibility	Full access	Unable to be accessed
Language	English	Non-English
Timeline	2019–2023	<2019

#### 2.3.3 Eligibility

In the eligibility stage, the titles, abstracts, and main contents of the remaining 32 articles were thoroughly examined to ensure that they fulfilled the criteria and were fit to be used in the current study to achieve the research objectives. A total of 14 articles were excluded because they were not based on empirical data or did not focus on the influence experienced by gig workers on digital platforms. Ultimately, 18 articles remained for analysis.

### 2.4 Data abstraction and analysis

This study used an integrative review, a review technique that analyzes and synthesizes diverse research designs together, which can be achieved by transforming one type into the other - quantizing qualitative data or qualitizing quantitative data [19,25]. This paper chose to qualify all selected data.

The process of developing appropriate themes was carried out based on thematic analysis. The first phase involved compiling data by carefully analyzing the 18 selected articles to extract statements or data that answered the research question. This was followed by creating meaningful groups via the coding method according to the nature of the data, converting raw data into useable data by identifying themes, concepts, or ideas for more connected data [19,26,27].

The process resulted in 12 main themes: information sharing, task allocation, entry into the market, transaction facilitation, shift and schedule patterns, quality of work, adjudication, control over the market, competition, market price control, employment status, and worker representation. A record was kept during the entire data analysis process, including thoughts, confusion, or any ideas associated with the data interpretation. Finally, the developed themes were adjusted accordingly to ensure their consistency.

## 3 Results

### 3.1 General findings and background of the studies

The analysis produced 12 main themes related to the influence of digital platforms on gig workers (Table 4). In terms of publication year, the reviewed studies span from 2019 to 2023, with three papers published in 2019 [[28], [29], [30]], one in 2020 [31], five each in 2021 [[32], [33], [34], [35], [36]] and 2022 [[37], [38], [39], [40], [41]], and four in 2023 [[42], [43], [44], [45]].

**Table 4:** Heatmap of the main theme.

Authors	IS	TA	EM	TF	SSP	QW	Adj	CM	Comp	MPC	ES	WR
Keith et al., 2019 (United States)					✔						✔	✔
van Slageren et al., 2022			✔									
Stephany et al., 2020 (United States)					✔				✔			
Mourelatos et al., 2022						✔						
P. Sun et al., 2023 (China)			✔		✔			✔	✔		✔	
Zhou & Pun, 2022 (China)	✔				✔					✔		✔
Han et al., 2021 (China)								✔				
Azzellini et al., 2021		✔	✔	✔		✔	✔		✔	✔		✔
Wang et al., 2023 (China)						✔						
He & Goh, 2022 (Vietnam)		✔										
G. Sun, 2023 (China)	✔	✔			✔	✔				✔	✔	
Nierling et al., 2023 (Germany)			✔		✔	✔	✔	✔	✔	✔		✔
Dokuka et al., 2022 (Russia)			✔		✔							
Burbano, 2021 (United States)						✔						
Rachmawati et al., 2021 (Indonesia)			✔		✔		✔			✔	✔	✔
Williams et al., 2021 (Australia)		✔	✔					✔		✔	✔	✔
Polkowska, 2019 (Poland)	✔	✔	✔	✔	✔			✔	✔	✔	✔	✔
Eskelund et al., 2019 (The Philippines)			✔									

Notes: IS: Information Sharing; TA: Task Allocation; EM: Entry of Market; TF: Transaction Facilitation; SSP: Shift and Schedule Pattern; QW: Quality of Work; Adj: Adjudication; CM: Control over Market; Comp: Competition; MPC: Market Price Control; ES: Employment Status; WR: Worker representative.

Within this date range, most of the published papers focused on China [34,41,43,44,45], followed by the United States with three papers [29,31,33]. Two papers conducted comparative studies between countries in Europe [32,40], while one paper discussed gig workers in digital platforms in a general context [39]. The remaining studies were conducted in Germany [42], Russia [37], Australia [36], Poland [30], Vietnam [38], Indonesia [35], and the Philippines [28], with at least one paper each.

### 3.2 Main findings

Digital platforms have brought about changes in the arrangement of gig work, which have influenced gig workers. This section discusses 12 main themes or transformations in digital platforms: information sharing, task allocation, entry into market, transaction facilitation, shift and schedule patterns, quality of work, adjudication, control over market, competition, market price control, employment status, and worker.

#### 3.2.1 Information sharing (IS)

Multiple studies highlighted how digital platforms facilitate information sharing between gig workers and clients to accommodate easy task allocation and completion, but they also enable invasive data collection, extensive monitoring of gig workers, and exploitation of gig workers [30,41,43]. Digital platforms have increased the necessity of information sharing within the industry and among gig workers and clients. While some information sharing is necessary, oversharing may negatively influence participants. In the e-hailing industry, information sharing with gig workers is considered insufficient, leading digital platforms to allow drivers to reject or cancel offered rides [30].

Data sharing with market intermediaries is seen as rampant and has negatively influenced gig workers. Despite promoting full freedom and autonomy, digital platforms record personal data on gig workers for high monitoring levels [43]. Gig workers perceive the manipulation of algorithms by digital platforms to benefit themselves as the root cause of an exploitative labor regime for the immoral pursuit of profits [41]. Gig workers also lack a full grasp of the system and calculation, such as knowing the role of fleet partners in Uber in Poland. They are unaware of the weekly commission charges on these partners despite being aware of the commission they need to pay to Uber. Moreover, gig workers have no full knowledge of the kind of contract they are bound by or who the other party is [30]. While information sharing for gig workers is seen to be insufficient, information sharing of gig workers is often financially exploitative to them.

#### 3.2.2 Task allocation (TA)

Digital platforms have streamlined the process of matching gig workers with clients, although challenges remain in some sectors, such as music [32]. The features in digital platforms have eased the task allocation process for gig workers in a short time, calculating the estimated delivery time and planning the delivery route, while gig workers face the arbitrary supervision of algorithmic management [43]. Digital platforms also serve as a medium for gig workers to promote their services and connect with clients. However, gig workers are expected to learn and develop new skills in creating and promoting personal brands [36]. Furthermore, certain digital platforms occupy a small, non-lucrative market segment, suggesting that the odds of gig workers getting work are weak, and digital platforms do not appear to be a viable way for gig performers to build careers [32].

In the music industry platforms, the fragmented demand side and the differing logic governing interactions with different types of buyers pose issues, as tasks cannot be efficiently allocated when different gig performers provide different genres of services [32]. On some digital platforms, gig workers are allowed to reject tasks or cancel accepted tasks. Nevertheless, gig workers do not overuse this opportunity; instead, it gives them a favorable opinion of their work and a sense of independence [30]. On the other hand, He and Goh [38] asserted that allocation policies in digital platforms should not be derived from static models to obtain better performance.

#### 3.2.3 Entry into market (EM)

Digital platforms offer low barriers to entry for gig workers from various backgrounds and cross-regional locations, but this decreases task opportunities since gig workers are oversupplied compared to the demand [30,36]. Digital platforms offer access to the job market, especially for international crowd workers, allowing them to participate in an otherwise closed market [42]. However, gig workers from the same country are hired two and a half times more, indicating that gig hiring is less likely to cross national boundaries and is often among countries with a common official language. Interestingly, cultural and institutional quality differences did not significantly affect gig hiring [40].

Digital platforms do not explicitly value academic and formal qualifications [42], and technology is easily navigated even by less IT-literate individuals [30], resulting in gig workers usually being high school graduates [35]. Digital platforms also give people with disabilities the opportunity to work and contribute to society [28]. The low barrier of job requirements was the main reason for becoming gig workers [35] and has encouraged female gig workers to join, as they experience a lack of job options or opportunities due to age and educational factors [37]. Consequently, the low requirements have lowered gig workers' expectations of working conditions or even standard workers' rights [30], allowing them to accept a temporarily disadvantageous situation [42].

Gig workers find the gig work arrangement better than regular job tenure or short-term contracts, albeit challenging [35]. They would continue in gig work because of higher income compared to other jobs, more control over their working schedule, and the need to take care of family responsibilities [44]. However, gig workers find that work opportunities have declined as many new gig workers enter the market [30,36]. This has also resulted in a churning effect where an increasing number of gig workers enter the market but leave after a few years to seek employment elsewhere [36]. Consequently, several big digital platforms have started to restrict the recruitment of new gig workers [35] and conduct more extensive evaluations before accepting gig performers, judging them by their musical quality and categorizing them accordingly [32]. However, digital platforms have not weakened the relationship between elite performers and their agents; instead, an agent's prestige increases with the exclusivity of their access to the client [32].

#### 3.2.4 Transaction facilitation (TF)

The transaction process facilitated by digital platforms simplifies the payment method and avoids the possible risks that gig workers may experience [30]. Digital platforms have developed features that facilitate payment and transactions within the platform itself. However, fully automated transactions are still impossible for certain industries, like music industries, as human intervention is still required [32]. For other industries like the e-hailing industry, cash payments are seen as only complicating the system and resembling the one applied in the traditional taxi industry [30]; hence, a full transition into online payment is expected. Furthermore, gig workers find that the online payment system improves their security since they do not have to carry cash, thereby avoiding the risk of robbery [30]. This has reduced the possibility of encountering incidents like robbery or fraud that would harm gig workers.

#### 3.2.5 Shift and schedule pattern (SSP)

Previous studies have shown that the flexibility of work arrangements has been the main reason for attracting the participation of gig workers. However, for the same reason of high participation, flexibility has decreased, and gig workers are expected to work more than usual [30,43].

Flexibility and autonomy have been the major causes for the participation of gig workers [42], especially for female gig workers [37]. This working arrangement works very well for gig workers whose spouses or partners are being laid off or need to work at home, as gig workers can easily alter their working arrangements due to the flexibility of gig work [31]. Nonetheless, gig workers have attained a consistent working time pattern [35], suggesting a thinning line of flexibility in gig work.

Digital platforms also reward or punish gig workers based on client evaluations. Gig workers will be punished if they fail to complete tasks within the specified time, such as having a certain amount of money deducted if the task is overdue and unable to click the task as completed in advance [43]. The number of tasks completed depends on whether gig work is regarded as the primary income for gig workers. Additionally, gig workers who view gig work as their regular job would create a working schedule and spend more time completing tasks [29].

Gig workers in e-hailing drive about 10 h daily, including weekends, significantly higher than standard working hours [30]. Gig workers perceive longer working hours and more tasks to lower their social support, psychological capital, and mental health; working more than 12 hours a day causes burnout among gig workers, affecting their work enthusiasm, sense of success, and physical well-being. Moreover, reducing gig workers' autonomy, the continuous occupation of a lifetime by work tasks, and the increasing workload through algorithmic management also affect their mental health [43] and their family lives [30]. Freedom and flexibility have declined as digital platforms become more demanding. Certain digital platforms calculate break time, and if gig workers appear offline for too long, the digital platforms send them alert messages. Rigorous digital surveillance blurs gig workers' work and social spaces and creates a work-as-life illusion, generating consent and attachment among gig workers [44]. Gig workers find the abusive and arbitrary management unjust and disrespectful, making them feel like they lose the kind of control over life a grown-up should have [41].

#### 3.2.6 Quality of work (QW)

Digital platforms prioritize the quality of work through algorithmic monitoring, yet this has severely influenced gig workers' financial income and social protection [29,42,44]. Various factors, such as gender, region, educational background, and computer literacy, influence the work quality of gig workers. Female and Asian gig workers show slightly lower performance in crowdsourced online gig work [39], while in the music industry, gig workers are sorted by popularity instead of user-generated measures of quality [32].

Digital platforms quantify gig workers' behavior by recording all details of their whereabouts, thereby supervising their work process and improving their service quality. However, this strict monitoring negatively affects gig workers, reducing their satisfaction and happiness, increasing emotional exhaustion, and lowering their work performance. Algorithmic management also directly influences gig workers' labor process, labor experience, and mental health [43], with digital platform statistics causing gig workers more stress than client expectations [42].

Interestingly, task duration weakens the effect of task reward on the quantity of contributors while strengthening the effects of task reward on the quality of contributors [45]. Furthermore, charitable giving by digital platforms increases gig workers' willingness to do extra work, and even sharing information on charitable giving alone increases the feeling of closeness toward digital platforms [33]. However, maintaining good quality work is difficult when gig workers do not possess the necessary skills or knowledge, and it takes a toll on the flexibility of working, thus affecting gig workers' mental health.

#### 3.2.7 Adjudication (Adj)

Previous studies have highlighted the responsibility of digital platforms to adjudicate matters between gig workers and clients. However, it is undeniable that the mediation process is often unfair and negatively impacts gig workers [35].

Digital platforms are first contacted to mediate between gig workers and clients when conflicts arise. However, this is not always the case, as certain gig workers have learned to differentiate and avoid tasks that have potential conflict because digital platforms usually advise gig workers not to pursue the dispute further [42]. Gig workers face several types of conflicts and risks, particularly in the e-hailing industry, where they are exposed to traffic accidents and street crimes. While digital platforms may provide insurance as a solution, they often have strict requirements and do not cover gig workers' vehicles, even if they are badly damaged or robbed during working hours [35].

Moreover, digital platforms accept complaints and poor performance ratings from clients without further investigation, reflecting the injustice towards gig workers when dealing with conflicts. This has led gig workers to raise their dissatisfaction by protesting in front of the platform companies' offices [35]. The importance of adjudication varies across industries, with the music industry presenting a different scenario. In this industry, clients demand adjustments where the factors are deemed complex, requiring specific and sometimes detailed logistical information with price ramifications, and digital platforms feel frustrated when bands are demanding on their clients [32]. Digital platforms in the music industry are intermediaries pressured to satisfy both parties.

#### 3.2.8 Control over market (CM)

Previous studies suggest that digital platforms are self-regulators in the market without government intervention [46]. This allows digital platforms to control the oversupply of gig workers and enables them to misuse and manipulate other platforms to exploit gig workers [44].

Gig workers recognize the necessity for digital platforms to have exclusive access to gig crowds from wealthy countries and protectionist measures so that they can negotiate high salaries and get tasks from recruitment instead of applying. The emergence of different markets within digital platforms, characterized by cheap labor and highly qualified and specialized project categories, limits the kinds of projects each gig worker can apply to. Despite not being seriously affected by it, gig workers strongly reject the cheap labor market [42], as a situation of lower demand and overflowing supply could happen.

Digital platforms have controlled the market by allowing mass participation of new gig photographers or hobbyists in the industry, exploiting them to provide below-cost photography [36]. This has led to decreasing tasks over the years, making gig photographers pessimistic about the prospects and income from photography. The influence of digital platforms on the local market landscape is evident, with foreigners working as gig workers for Uber in Poland negatively impacting the local Uber gig drivers by reducing their usual task volume. Additionally, while it may seem that gig workers can work anytime they want, they work based on the high demand they frequently get according to the time of day, meaning market demand impacts when they work and earn the most [30].

The control of the market by digital platforms enables them to misuse other social media platforms and entrap gig workers into a broader mediated labor control system beyond its environment [44], affecting the working flexibility that was once promoted. However, digital platforms in the crowdsourcing industry do not seem to control the market completely. When gig workers sign up for tasks, they are introduced to vendors hiring them for services. As the reputation of the vendors improves, the willingness to participate in the task-based market or gig work gradually decreases. In contrast, the willingness to participate in the employment market gradually increases [34]. This suggests that gig workers are more attracted to leaving the market and pursuing conventional working arrangements.

#### 3.2.9 Competition (Comp)

Gig workers face substantial challenges regarding competition within the platform economy. They must maintain ratings, improve service quality, and widen their range of skills, often adhering to lower rates to secure work and earn income [31,42].

Digital platforms feature rating systems and allocate tasks to gig workers based on task success scores, making a competitive project history crucial for gig workers [42]. In the music industry, digital platforms conduct quality judgments on gig workers to categorize their forte and genre of performances, which can limit their access to tasks [32]. Maintaining ratings is challenging for gig workers [42]. In some cases, like those of foreign Uber drivers in Poland, their lack of language proficiency and local knowledge can spoil the company's reputation among clients. Uber's surge pricing system, intended to motivate gig drivers to move to higher-demand areas, does not always work correctly. It is often disabled when gig workers arrive, making it not worth pursuing [30].

Furthermore, the supply of gig workers often exceeds the available tasks, forcing them to bid on more jobs, often below their target salary rates or minimum wage, to secure work [31,42]. Gig workers are also compelled to diversify and apply to different types of tasks to increase the number of tasks they can bid on, which affects their financial income and working hours [31]. The intense competition within the industry has led digital platforms to forbid gig workers from applying for tasks on other platforms [44], a situation made possible by the lack of interference from authorities to monitor and regulate the market.

#### 3.2.10 Market price control (MPC)

Digital platforms' control over pricing methods has negatively influenced gig workers' financial income stability [32,35,36]. Pricing on digital platforms is usually calculated using algorithms. It is not pre-determined, leading to surge pricing in the e-hailing industry, where rides in a particular area can be more expensive due to higher demand and limited supply [30]. While higher income reflects higher job satisfaction and work enthusiasm for gig workers, positively affecting their psychological capital and mental health [43], their earnings are often unstable and sometimes fall below the minimum wage. This situation is exacerbated during pandemics due to large-scale social restrictions [35]. Gig workers are concerned about the longer-term economic slowdown, as a study by Stephany et al. (2020) shows that less than 40 % of gig worker respondents have healthcare and more than a few weeks of savings. In the music industry, gig musicians display their desired fees while clients can publicly post them, but both tend to be very low [32].

Although digital platforms provide more developed price comparison mechanisms, they also allow intermediaries to interfere. Digital platforms enable intermediaries to pursue offline profit-making strategies by not displaying prices to clients and negotiating high prices with clients, keeping the difference as a concealed commission. Creative-oriented platforms cannot rely on algorithms to determine prices and instead depend on interpersonal negotiation, often resulting in low-paid or underpaid engagements with a 'take-it or leave-it' approach to pricing. This could be one of the factors preventing such digital platforms from progressing into higher-value live music work [32].

While digital platforms increase demand, they also erode the value of services, as seen in the photography industry. Clients struggle to distinguish the quality of work, forcing professional gig photographers to devalue their work. The rise in the number of individuals taking photos with decent-quality cameras puts pressure on the price of professional products and services. The value of products and services is further eroded by the low understanding of value among new professionals who undercut previously regular market rates, and even previously high levels of income have been substantially eroded and will never return [36]. This low income cannot support gig workers without working more than 40 h a week [30].

Access to tasks on digital platforms has become a bottleneck, leading to lower pay rates and prompting gig workers to demand regulations to end price dumping [42]. In response to the affected income, gig workers also engage in various forms of protest, such as work stoppages, collective logging out, open letters, and demonstrations. Digital platforms are seen to dwindle remuneration, reduce subsidies, decrease orders, and increase commission rates and costs [41]. Moreover, digital platforms do not provide adequate 'reward versus effort' or 'fair compensation' for investments in time, costs, and creative skills, forcing gig workers to make their investments [36].

#### 3.2.11 Employment status (ES)

Gig workers suffer from work identity and classification issues when working on digital platforms, leading to their separation from social protection benefits and job insecurity [35,44]. The work identity of gig workers is often inconsistent with the current environment, leading to tension [43]. Different perceptions among gig workers regarding whether they consider their gig work as 'work' are reflected in their earning satisfaction [29]. Keith et al. [29] suggested that gig workers who consider MTurk as a job do so because they spend time doing it and are paid for it, while those who do not consider it their 'work' often report not making enough money for it to be considered as such. The 'partner' status of gig workers means they are not entitled to form or join a trade union because they are not considered workers [35].

Some gig workers have experienced a shift in employment relations. A respondent in the study by P. Sun et al. [44] recounted how he was first employed as a courier. However, his contract and monthly salary were canceled, shifting him from an employee to an outsourced laborer. Digital platforms often prefer full-time gig workers over part-time ones because the latter usually enjoy a flexible working schedule. At the same time, the former are required to work at least 8 hours daily and have a fixed schedule [44]. However, even full-time gig workers are paid based on fulfilled tasks instead of log-on hours, leading to deep job insecurity [35,44].

Despite this, some gig workers are convinced of the stability and security of gig work, although they are unsure how long they will be allowed to continue [30]. Technology also undermines gig photographers' identity as creative artists [36]. Gig workers realize that certain problems stem from their status not being formally regulated. However, they do not see themselves doing gig work in the future, making them ignorant of the legal and formal issues concerning their status. While they may want protection, they are not ready to contribute more financially [30].

#### 3.2.12 Worker representative (WR)

Gig workers cannot form unions due to their worker classification, further distancing them from their rights as laborers [35,42]. Labor unions in gig work are absent, and gig workers can only interact through official community forums, but their isolated nature of work means they never meet each other [42]. Although not entitled to form a union, gig workers still hold occasional meetings among geographically based communities, which support each other in their work activities, such as helping each other when involved in an accident, sharing daily information and updates, and collecting donations for those in need. While these communities are seen as helpful, they are not strong enough to strengthen gig workers' status [35].

Similarly, the photographic industry is not unionized, but gig photographers find the existence of a professional community necessary [36]. However, some gig workers have no interest in joining a union or receiving their protection even if given the opportunity. Moreover, the presence of foreign gig workers makes it impossible to discuss any community of interest among gig workers [30].

On the other hand, digital platforms also attempt to connect with gig workers by attending their community gatherings, which some gig workers find hopeful, while others regard them with skepticism [35]. Digital platforms like MTurk provide message board platforms for gig workers to voice complaints, socialize, and share information about their work activities. MTurk gig workers who state MTurk as their primary source of income are most likely to read the messages, while those who do not state it as their primary source of income are most likely to post the messages [29].

Social media channels, such as WeChat for Didi drivers in China, are significant in gig workers' solidarity and collective mobilization. WeChat's features of 'one-to-many acquaintances' and 'limited-group orientation' allow users to organize and coordinate close-knit group activities that address issues of shared concern. These groups form a more extensive network connecting most drivers in a broader geographical area [41].

Gig workers also connect among themselves when hanging out to fill the boredom of waiting for tasks. Apart from being a platform to voice grievances, social media groups serve as information-sharing platforms for gig drivers to update each other on traffic police and traffic conditions. Grievances shared include the quantity and quality of orders received, opaque order-dispatching and service-point mechanisms, arbitrary management practices, gig workers' outrageous or unusual behavior, and excessive work intensification. Gig workers believe that collaborating can bring about change and improvement, as previous issues have been solved when raised, and action is necessary to address their grievances. Gig drivers who used to be in lower management usually initiate the discussion, which often reaches offline meetings. However, gig drivers' participation is not compulsory and depends on their evaluation of the feasibility, safety, and necessity of collective action [41]. In industries with unions, like music, the musician union encourages members to negotiate contracts that include specific arrival, performance, and packing-up times [32], which would benefit gig musicians.

Although gig workers have developed unofficial communities to communicate and share concerns, their deteriorating and unregulated working conditions call for establishing a more substantial body or organization to fight for them.

## 4 General discussion

The reviewed evidence suggests that digital platforms positively influence gig workers in several aspects, such as offering low barriers to entry [28,35,37,42]. However, these low entry barriers lowered gig workers' expectations of working conditions and rights [30]. Moreover, the high participation of gig workers resulting from low entry barriers eventually creates a churn effect [30,36]. Low barriers to entry also affect local gig workers' reputations when gig workers lack local knowledge to conduct the tasks [30]. Gig workers experience decreasing task opportunities when they are highly involved -- including foreign gig workers -- in the market [30,42]. This situation has also resulted in gig workers accepting low target salaries aligned with low demand for their services [31,42]. Therefore, gig workers find protectionist measures beneficial to limit participation and protect prices [36,42].

The decreasing opportunities for tasks stem not only from low barriers to entry. Competition between gig workers increases as different markets emerge within digital platforms [42]. In certain industries, gig workers' services are devalued because skills are easily imitated through advanced technology and the high participation of non-professional gig workers [36]. Consequently, gig workers are encouraged to develop new skills [36] to match tasks requiring various skills [32]. However, diversifying skills takes much time and affects their working hours [31].

Other positive influences digital platforms bring to gig workers include flexible working arrangements that benefit gig workers who have other arrangements to attend to Ref. [31], facilitated transactions that secure and lessen the possibility of risks for gig workers [30], and a straightforward process of matching gig workers with tasks and clients [43]. Gig workers are allowed to reject tasks when there is a lack of information about them, which could confuse them. Nevertheless, gig workers have to strategize where and when there will be demand, which distances them from the flexibility of working [30].

On top of that, gig workers' data is harvested and then used to closely monitor them, distancing them from autonomy and flexibility [43,44]. Although algorithmic surveillance improves service quality [43], it results in work-life imbalances, reduces service quality due to decreased work satisfaction and enthusiasm, and makes them feel like they lose control of their life [41,43,44]. Digital platforms use this information to exploit gig workers [41]. Gig workers are forbidden from applying for tasks on other digital platforms, affecting their financial income [44]. The intermediaries in digital platforms limit information like fees displayed to earn extra commissions [32]. In addition, digital platforms manipulate remuneration, subsidies and decrease orders while increasing commission rates and costs for gig workers, which affects their income [41].

Digital platforms mediate issues between gig workers and clients and assist them with solutions [32,35,42]. However, fulfilling requirements to earn solutions is rather strict [35]. In addition, pricing in digital platforms uses the 'take-it or leave-it' approach, so gig workers are expected to accept the low desired fees [32]. These unstable earnings are concerning given that gig workers do not have healthcare and enough savings, hence forcing them to work more than usual, which then affects their mental health [30,31,35,43]. The surge pricing held to increase prices did not work because it was often turned off before they could get it [30].

Furthermore, maintaining ratings and clients' expectations in digital platforms is challenging for gig workers and often stresses them out [42]. Gig workers could be financially punished, suspended, and lose task allocation due to low ratings or incomplete tasks [32,42,43]. On top of that, client complaints are often received without further investigation, which is unjust to gig workers [35].

Another challenge that gig workers experience is that their status as partners often stresses them out and distances them from forming a union [35,43]. Gig workers are expected to work like full-time employees but are paid part-time, which affects their financial income and flexible working arrangements [44]. The most digital platforms could do was to reach out through fieldwork meetings or set up online message boards [29,35]. It could be seen that a union in the gig music industry helps gig performers negotiate contracts [32]. Instead, gig workers create unofficial communities on social media platforms to share concerns and issues among themselves, although participation is not compulsory [35,41]. These unofficial communities are weak enough to strengthen their status [35]. On top of that, forming a union is almost impossible due to the disinterest of gig workers, the existence of foreign gig workers, and the refusal to make financial contributions to it [30,36]. These findings align with prior research that suggests that gig workers are distanced from incentives and benefits commonly given to conventional workers [11].

These findings suggest that digital platforms positively influence gig workers through low barriers to entry [28,35,37,42], easy task allocation matching [43], and adjudication in handling issues [35], which allows them to make additional income according to their working arrangements. However, gig workers also face substantial challenges, including a lack of benefits and protections [35], algorithmic control and surveillance [44], and downward pressure on wages [41]. These findings align with prior research highlighting digital platforms' complex and contradictory influence on gig workers [10].

The implications of this review's findings are significant for sustainable and inclusive economic development in the digital age. The review underscores the importance of collective organizing and advocacy to secure better working conditions and protections for gig workers [41]. For digital platforms, the findings suggest a need to re-evaluate practices around data collection, algorithmic management, and worker classification and prioritize gig workers' well-being and rights. For policymakers, the review highlights the necessity of stronger labor regulations and social safety nets to address the unique challenges faced by gig workers and ensure the benefits of technological innovation in the platform economy are shared more equitably.

## 5 Recommendations

The findings and systematic review process of the present study have led to several recommendations that may be helpful for future research.

The themes developed from this systematic literature review are information sharing, task allocation, entry into market, transaction facilitation, shift and schedule pattern, quality of work, adjudication, control over market, competition, market price control, employment status, and work representation. These themes are consistent with the study of Heeks et al. [46] on digital platforms and institutional voids in developing countries. The study described how platform companies, as institutional entrepreneurs, create new or impact existing institutions that later fill in the shortcomings of institutions that a market could have. Information, aggregation, management, and regulation are the institutional voids that are filled or replaced by digital platforms. While the study utilizes institutional voids to explain the influence of digital platforms on markets, this concept could be expanded further to explore the influence of digital platforms on gig workers.

The results suggest several implications and areas for further investigation. The findings underscore the importance of collective organizing and advocacy for gig workers to secure better working conditions and protections [41]. The review highlights a need to re-evaluate practices around data collection, algorithmic management, and worker classification for digital platforms. Finally, policymakers should consider strengthening labor regulations and social safety nets for gig workers.

Another critical area for future research is the role of gender, race, and other demographic factors in shaping the experiences of gig workers on digital platforms. Previous studies have suggested that women and racial minorities may face unique challenges and barriers in the gig economy (e.g., Refs. [37,39]), and more research is needed to understand and address these inequities.

Future research should also consider the broader societal and economic implications of the growth of the gig economy and the increasing influence of digital platforms. This could include studies on the impact of gig work on traditional employment relationships, the role of the gig economy in exacerbating or alleviating income inequality, and the potential for gig work to provide opportunities for marginalized populations.

In conclusion, while this systematic review provides valuable insights into the influence of digital platforms on gig workers, there is still much to be learned about this rapidly evolving and complex topic. By addressing the gaps and limitations identified in this study and pursuing the recommended areas for future research, scholars can contribute to a more comprehensive and nuanced understanding of the gig economy and its impact on workers, platforms, and society.

## 6 Limitation

The present study's systematic literature review has several limitations that can be proposed for future research.

It is important to note that this study has no regional focus, given that related published papers are scarce and the topic is relatively new and unexplored in many countries. This presents an opportunity for more focused studies in the future, with a regional emphasis on developing countries, third-world countries, East or West countries, or the role of languages and culture in shaping the relationship between gig workers and digital platforms.

Furthermore, this study does not focus on a distinctive industry of gig workers. While gig workers exist in industries utilizing digital platforms, there are apparent differences in operational practices between them. Therefore, further research should explore the influence of digital platforms on gig workers in specific industries, such as crowdsourcing gig work, creative gig work, or transportation and delivery gig work.

Additionally, this study only utilizes journals and articles written and published in English. The researchers believe this limitation may constrain the available information, as exploring journals in other languages or focusing on a country with a common language could provide a more comprehensive overview of this field of study.

Future research should also consider employing a mixed-methods approach, combining qualitative and quantitative data to provide a more comprehensive understanding of the influence of digital platforms on gig workers. Qualitative methods, such as interviews and focus groups, could provide rich insights into gig workers' lived experiences and perspectives. In contrast, quantitative methods, such as surveys and platform data analysis, could help identify broader patterns and trends.

## 7 Conclusion

The recent literature on the influence of digital platforms on gig workers provides a foundational understanding of the experiences of gig workers in the digital platform economy. Through a systematic review, this research identified twelve main themes that represent the key ways in which digital platforms influence gig workers: information sharing, task allocation, entry into market, transaction facilitation, shift and schedule patterns, quality of work, adjudication, control over market, competition, market price control, employment status, and worker representation. These themes offer valuable insights into the complex and multifaceted nature of the gig economy and its impact on workers.

The findings of this review suggest that digital platforms have positive and negative influences on gig workers. On the one hand, digital platforms offer low barriers to entry, facilitate task allocation and transaction processes, and provide opportunities for flexible work arrangements. These factors can enable gig workers to access new sources of income and exercise greater control over their work schedules. On the other hand, gig workers face significant challenges, including a lack of social protections and benefits, algorithmic control and surveillance, intense competition, and downward pressure on wages. These challenges can lead to financial insecurity, work-life imbalances, and mental health issues for gig workers.

Furthermore, the review highlights the power asymmetries between gig workers and digital platforms, with platforms exercising significant control over the market, pricing, and working conditions. Gig workers often lack the bargaining power and collective representation necessary to advocate for their rights and interests, leaving them vulnerable to exploitation and unfair treatment. While some gig workers have formed unofficial communities and engaged in collective action, these efforts are often limited in scope and impact.

The findings of this review have important implications for gig workers, digital platforms, and policymakers. The review underscores the need for greater collective organizing and advocacy for gig workers to secure better working conditions and protections. For digital platforms, the review highlights the need to re-evaluate practices around data collection, algorithmic management, and worker classification and prioritize the well-being and rights of gig workers. For policymakers, the review suggests the need for stronger labor regulations and social safety nets to address the unique challenges faced by gig workers.

Overall, this systematic review contributes to a growing body of literature on the gig economy and its impact on workers. This review provides a valuable foundation for future research and policy debates by synthesizing the existing research and identifying key themes and challenges. As the gig economy continues to evolve and expand, researchers, platforms, and policymakers must work together to develop strategies and solutions that prioritize the well-being and rights of gig workers. Only by addressing the underlying power imbalances and inequities in the gig economy can we ensure that the benefits of digital platforms are shared more equally and that gig workers can thrive in this new world of work.

## CRediT authorship contribution statement

**Farah Diba Almayanda Alauddin:** Writing – review & editing, Writing – original draft, Methodology, Investigation, Formal analysis, Conceptualization, Data curation. **Aini Aman:** Writing – review & editing, Supervision, Conceptualization, Project administration, Validation. **Mohd Fahmi Ghazali:** Writing – review & editing, Writing – original draft, Supervision, Investigation, Conceptualization, Funding acquisition, Methodology, Project administration, Validation. **Sity Daud:** Writing – review & editing, Supervision, Conceptualization, Validation.

## Ethics declarations

This systematic literature review did not involve any primary data collection from human participants, animal subjects, or clinical trials. Therefore, ethics committee approval was not required. The study analyzed publicly available policy documents and published research papers following established systematic review protocols.

## Data and code availability statement

Data will be made available on request. For requesting data, please write to the corresponding author.

## Declaration of generative AI use

The authors declare that no artificial intelligence tools, including generative AI, were used in the writing or editing process of this manuscript.

## Funding statement

This work was supported by the Faculty of Economics and Management, Universiti Kebangsaan Malaysia.

## Declaration of Competing Interest

The authors declare that they have no known competing financial interests or personal relationships that could have appeared to influence the work reported in this paper.
